# COVID-19 Vaccine Acceptance Among Rural Appalachian Healthcare Workers (Eastern Kentucky/West Virginia): A Cross-Sectional Study

**DOI:** 10.7759/cureus.16842

**Published:** 2021-08-02

**Authors:** Tuong Vi C Do, Sanjana Thota Kammili, Michael Reep, Lauren Wisnieski, Subramanya shyam Ganti, Jayaramakrishna Depa

**Affiliations:** 1 Internal Medicine Residency Program, Appalachian Regional Healthcare, Harlan, USA; 2 Internal Medicine Residency Program, Appalachian Regional Healthcare, Whitesburg, USA; 3 Public Health and Research, Lincoln Memorial University, Harrogate, USA; 4 internal Medicine Residency Program, Appalachian Regional Healthcare, Harlan, USA

**Keywords:** covid 19 vaccine, acceptability of vaccine, healthcare worker, incentives, vaccine hesitancy

## Abstract

Introduction

The success of a vaccination program is dependent on vaccine efficacy and the number of people vaccinated. Healthcare workers are the first to receive the COVID-19 vaccine based on CDC phase 1a recommendations and are a point of contact for information for patients, so they must be well-educated on common misconceptions about the vaccine.

Objective

To identify acceptance/refusal rates of COVID-19 vaccine, reasons for refusal, and to understand the impact of demographics, work environment, and comorbidities on vaccine acceptance.

Methods

A cross-sectional study of 1076 healthcare employees in Rural Appalachian utilizing electronic and paper-based 12 question surveys from December 10, 2020, through December 20, 2020, followed up to April 2021.

Results

Within our study, 52.3% of our healthcare workers would accept vaccination with higher age, male gender, physicians, and those who receive annual flu vaccines more likely to accept vaccination. The most common reason for refusal was unknown side effects (88.5%). The second reason for refusal at 33.5% was waiting for someone else to take the vaccine first. In February 2021, the percentage of our healthcare workers who were vaccinated was 48%, which then increased to 55% in March 2021. By April 2021, the vaccination percentage of our healthcare workers reached 59%.

Conclusions

In order to predict how the public percentage of vaccination would be, healthcare workers need to address concerns about side effects from the vaccines and encourage the public to get the vaccines since healthcare workers themselves had already received the vaccines and can educate the patients on how they did after getting the shots.

## Introduction

In 2019, a new virus coronavirus 2019 (COVID-19), also known as severe acute respiratory syndrome coronavirus 2 (SARS-CoV-2), first appeared in China piquing the interest of many scientists and healthcare workers across the world. This virus has been responsible for a worldwide pandemic that began in March 2020. At the time of this study, COVID-19 has infected more than 86.1 million people including 20.9 million cases within the United States (US). There have been 1.86 million deaths worldwide including 354,000 deaths in the US [1]. In the rural Appalachia area involving Eastern Kentucky and West Virginia, there have been 491,980 cases of COVID-19 with 5,894 deaths [2,3].

Billions of dollars are being invested into research on the pathogenesis, treatment, and prevention of COVID-19. For prevention, at least 90 proposed vaccines were developed in hopes to provide immunity [4]. In November 2020, three pharmaceutical companies announced that their vaccines were in phase 3 of clinical trials and released preliminary efficacy rates to obtain emergency Food and Drug Administration (FDA) approval. Two vaccines were shown to be greater than 95% effective while the other vaccine was greater than 70%. In December 2020, the FDA authorized two vaccines for COVID-19, namely the Pfizer-BioNTech COVID-19 Vaccine and Moderna COVID-19 Vaccine [5]. By February 2021, the FDA approved Johnson and Johnson COVID-19 vaccine.

As with any vaccine, there is hesitancy in its acceptance. For example, the Flu vaccine, which was developed in 1940, had a low acceptance rate in the general population (48.4%) during the 2019-20 season in the US according to the Centers for Disease Control and Prevention (CDC). Among healthcare workers, the acceptance rate was higher at 80.6% [6]. With the COVID-19 vaccine being a new mRNA vaccine, the invention of a vaccine at such a rapid pace will have greater resistance to receiving the vaccine. An mRNA vaccine is built on the principle that the vaccine introduces an mRNA sequence that codes for a specific antigen of a disease within the body activating the immune system. Now that the immune system is activated, once exposed to that disease, the immune response would be quicker and more effective. Reasons for refusal could be related to quality control, side effects, fear of getting COVID-19, and efficacy of the vaccine. Many people are waiting for others to get the vaccine first [7-11].

The CDC has developed recommendations on phases of vaccination in early December 2020. Per CDC recommendations, healthcare personnel and residents of long-term care facilities should be offered the vaccine first (phase 1a), followed by essential frontline workers and people aged 75 years and older (phase 1b). The CDC further goes on to identify those included in phase 1c of vaccination with a note that recommendations will be expanded to include more groups as availability increases [12].

The objective of this study was to determine the percentage of healthcare workers in a large healthcare system located in rural Appalachia who is willing to receive the vaccine and to understand the impact of demographics, work environment, and comorbidities on vaccine acceptance. We also identified reasons for the refusal of the vaccine. Healthcare workers are responsible for educating patients on the vaccine, and their opinions play a crucial role in vaccine acceptance by the public [10-12]. At the time of our study, there were 1210 cases of COVID-19 within our healthcare system.

## Materials and methods

Data collection

**Figure 1:**
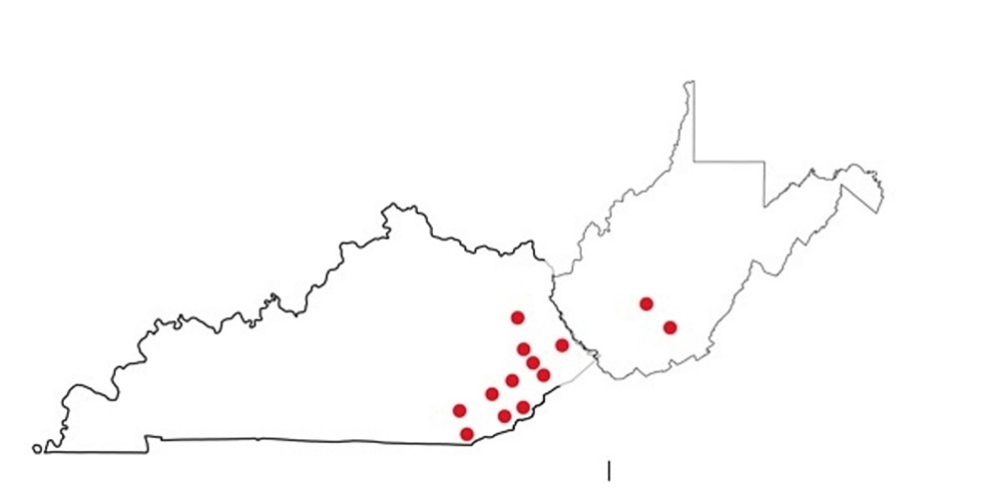
Rural Appalachia of the 13 healthcare systems

Data were collected from 13 healthcare facilities in Rural Appalachia as seen in Figure 1. All facilities were within the Appalachian Regional Healthcare network and are primarily located in Eastern Kentucky with two facilities located in West Virginia. Data collection took place from December 10, 2020, through December 20, 2020, prior to the initial round of vaccine administration. Our sample size was 6000 people employed within our healthcare system. A voluntary anonymous self-administered 12-question survey was utilized to collect the data. The questions were determined by the co-authors after referencing previous similar studies [7-11,13,14]. All co-authors agreed on the survey contents, which included questions on demographics, vaccine acceptance, comorbidities, reasons for refusal, sources of information on vaccine, etc.

The survey as seen in the Appendix first asked responders if they were willing to take the COVID 19 vaccine and then based on the response of no, they then had a further question asking why. When selecting options on why they were refusing, they were able to select multiple responses. We then asked if responders ever had COVID, and what was their exposure to COVID at work. Because we asked questions dealing with age, gender, and occupation along with hospital site, we did have responders who just exited the survey and did not complete it. Others made up an age of 0 or 99 because they did not want to be identified. We had a total of 1156 responses, but after the exclusion criteria as explained in Table 1, our total validated responses were 1076. We decided to exclude those who did not complete the survey in case they decided to retake the survey.

**Table 1:** Inclusion and exclusion criteria of responders

Inclusion Criteria	Exclusion Criteria
Those who gave consent to answer the survey and completed all 13 questions (1076).	Those who did not consent to answer the survey (9).
	Those who consented but did not provide any responses to the questions (2)
	Those who stopped the survey after the first question (5).
	Those who stopped the survey after the second question (3).
	Those who skipped question #13 (6).
	Those who skipped the demographic questions (55).

The survey was primarily administered electronically utilizing Survey Monkey. With Survey Monkey software, we marked all questions required in order to get complete responses. It also had a feature to allow one to take the survey once based on their IP address in order to avoid multiple responses from the same person. Because our demographic questions were placed before the question of who gets the flu vaccine annually, we wanted to exclude those responses because we wanted to compare the vaccine acceptance rate between the flu and COVID vaccines. An email with the link to the survey was sent via work email to all healthcare workers on the first day of the study. A repeat reminder email was sent five days later. A link accessing the survey was also posted on the internal hospital website as well as the corporations’ Facebook, which was only accessible to current employees. Responders to the survey included all those hired under our healthcare system including those working in the ER, the hospital itself, and on the ambulatory side that is associated with the hospital. However, this survey was conducted during the peak COVID census, so some responders were working from home. Additional responses for those who have not taken the online survey were obtained in paper format from two hospitals where the investigators worked. Participants provided informed consent prior to beginning the survey.

Given the nature of data collection and the information collected, this study was exempted by Appalachian Regional Healthcare Institutional Review Board (IRB).

Statistical analysis

All data cleaning and analyses were performed using Stata 14.2 (StataCorp 2015, College Station, TX). Categories of categorical variables with sparse sample sizes were combined for statistical analysis purposes. Chi-square and independent t-tests were used in descriptive analyses to assess what factors were associated with vaccine acceptance. Reasons for vaccine refusal among those with no acceptance of the vaccine were descriptively assessed. Confidence intervals for proportions were calculated with the exact method using the ‘cii’ command.

Mixed logistic regression was used to assess what factors were significantly associated with vaccine acceptance. Eligible variables for inclusion in the model included age, gender, race, occupation, facility type (hospital, ambulatory, emergency, other), comorbidities (hypertension, diabetes, chronic obstructive pulmonary disease [COPD], asthma, or congestive heart failure [CHF]), comorbidity count (sum of comorbidities 0-5), source of COVID-19 vaccine information (TV news, internet, academic journals, newspaper, discussion with coworkers, CDC, health department, or social media), had a COVID-19 positive test, level of exposure to COVID-19 patients (working with COVID-19 daily, weekly, monthly, or never), and whether they receive the yearly flu vaccine.

Backward elimination at P<0.05 was used to select variables in the final model. A random intercept for the location was included in the model to adjust for shared variance attributed to working at the same facility. Linearity of the association between continuous variables (i.e., age) and vaccine acceptance in the log odds scale was assessed using lowess smoothing curves. Variables that did not meet linearity transformations were transformed (i.e., square root or log transformation). Multiple comparisons between levels of categorical variables with more than two categories were adjusted for using Bonferroni adjustment.

## Results

In total, 1076 participants completed the survey out of approximately 6000 employees. Our ratio of female to male healthcare workers is estimated to be a 2:1 ratio based on the total of employees employed in our healthcare system. Overall, 52.3% of participants accepted the vaccine. Descriptive analyses of the variables including the demographics of the total population and the subgroups of those who accepted versus declined the vaccine are presented in Table 2. The average age (mean ± sd) was significantly (P < 0.001) higher among those who accepted the vaccine (44.5 ± 12.4 years) compared to those who rejected it (38.5 ± 11.5 years).

**Table 2:** Descriptive statistics of COVID-19 vaccine acceptance in a healthcare worker in rural Appalachia among 13 facilities (N=1076) *Proportion within total sample. ^†^Includes physicians, anesthesiologists, and Certified Registered Nurse Anesthetists (CRNAs). ^‡^Includes administration, housekeeping, IS, IT, maintenance, and other miscellaneous positions. ^§^Participants can select multiple answers, so the total number does not add up to 1076. ^||^Calculated using Fisher’s exact test. ^¶^Missing data: race (n=12), occupation (n=22), facility (n=16), comorbidity (n=54). Proportions calculated out of non-missing data. RN: registered nurse, NP: nurse practitioner, COPD: chronic obstructive pulmonary disease, CHF: congestive heart failure, CDC: Center for Disease Control and Prevention.

Demographics	Accepted vaccine		Declined vaccine		Total		Chi-square (df)	p-value
	n	P (95% CI)	n	P (95% CI)	n	P (95% CI)^*^		
Gender							24.9 (1)	<0.001
Male	144	67.6 (60.9–73.8)	69	32.4 (26.2–39.1)	213	19.8 (17.5–22.3)	-	-
Female	419	48.6 (45.2–51.9)	444	51.5 (48.1–54.8)	863	80.2 (77.7–82.5)	-	-
Race^¶^							0.79 (1)	0.37
White	525	52.0 (48.9–55.2)	484	47.9 (44.8–51.1)	1009	94.8 (93.3–96.1)	-	-
Non-White	32	58.2 (44.1–71.3)	23	41.8 (28.7–55.9)	55	5.2 (3.9–6.7)	-	-
Work environment occupation¶							27.8 (4)	<0.001
RN, NP, and nurse externs	144	52.4 (46.3–58.4)	131	47.6 (41.6–53.7)	275	26.1 (23.5–28.9)	-	-
Physicians^†^	52	82.5 (70.9–90.9)	11	17.5 (9.1–29.1)	63	6.0 (4.6–7.6)	-	-
Allied health	145	46.6 (41.0–52.3)	166	53.4 (47.7–59.0)	311	29.5 (26.8–32.4)	-	-
Pharmacists and pharmacy technicians	32	58.2 (44.1–71.3)	23	41.8 (28.7–55.9)	55	5.2 (4.0–6.7)	-	-
Other^‡^	185	52.9 (47.5–58.2)	165	47.1 (41.8–52.5)	350	33.2 (30.4–36.1)	-	-
Working with COVID-19 patients							7.78 (3)	0.051
Working with COVID-19 patients daily	164	49.1 (43.6–54.6)	170	50.9 (45.4–56.4)	334	31.0 (28.3–33.9)	-	-
Working with COVID-19 patients weekly	88	47.6 (40.2–55.0)	97	52.4 (45.0–59.8)	185	17.2 (15.0–19.6)	-	-
Working with COVID-19 patients monthly	41	48.8 (37.7–60.0)	43	51.2 (40.0–62.3)	84	7.8 (6.3–9.6)	-	-
Not working with COVID-19 patients	270	57.1 (52.5–61.6)	203	42.9 (38.4–47.5)	473	44.0 (41.0–47.0)	-	-
Facility type^§^								
Hospital	370	51.1 (47.4–54.8)	354	48.9 (45.2–52.6)	724	68.3 (65.4–71.1)	1.91 (1)	0.17
Ambulatory	123	54.9 (48.1–61.5)	101	45.1 (38.5–51.9)	224	21.1 (18.7–23.7)	0.64 (1)	0.43
Emergency	60	53.1 (43.5–62.5)	53	46.9 (37.5–56.5)	113	10.7 (8.9–12.7)	0.02 (1)	0.9
Other	83	63.8 (55.0–72.1)	47	36.2 (27.9–45.0)	130	12.3 (10.3–14.4)	7.59 (1)	<0.001
Comorbidities^§¶^								
Hypertension	194	60.2 (54.7–65.6)	128	39.8 (34.4–45.3)	322	31.5 (28.7–34.4)	11.47 (1)	0.001
Diabetes	66	50.0 (41.2–58.8)	66	50.0 (41.2–58.8)	132	12.9 (10.9–15.1)	0.36 (1)	0.55
COPD	19	59.4 (40.6–76.3)	13	40.6 (23.7–59.4)	32	3.1 (2.2–4.4)	0.64 (1)	0.43
Asthma	61	54.5 (44.8–63.9)	51	45.5 (36.1–55.2)	112	11.0 (9.1–13.0)	0.21 (1)	0.65
CHF	1	12.5 (0.3–52.7)	7	87.5 (47.3–99.7)	8	0.8 (0.3–1.5)	5.16 (1)	0.03||
None	280	49.4 (45.2–53.6)	287	50.62 (46.4–54.8)	567	55.5 (52.4–58.6)	4.79 (1)	0.03
Other								
Will recommend to family								
Yes	545	95.1 (93.0–96.7)	28	4.9 (3.3–7.0)	573	53.3 (50.2–56.3)	899.7 (1)	<0.001
No	18	3.6 (2.1–5.6)	485	96.4 (94.4–97.9)	503	46.7 (43.7–50.0)	-	-
Take flu vaccine yearly								
Yes	536	58.1 (54.8–61.3)	387	41.9 (38.7–45.2)	923	85.8 (83.5–87.8)	86.0 (1)	<0.001
No	27	17.6 (12.0–24.6)	126	82.4 (75.4–88.0)	153	14.2 (12.2–16.5)	-	-
Tested positive for COVID-19								
Yes	38	37.3 (27.9–47.4)	64	62.7 (62.6–72.1)	102	9.5 (7.8–11.4)	10.3 (1)	<0.001
No	525	53.9 (50.7–57.1)	449	46.1 (42.9–49.3)	974	90.5 (88.6–82.2)	-	-
Source of COVI9-19 vaccine information^§^								
TV (news)	311	50.3 (46.3–54.3)	307	49.7 (45.7–53.7)	618	57.4 (54.4–60.4)	2.33 (1)	0.13
Internet	365	52.4 (48.7–56.2)	331	47.6 (43.8–51.3)	696	64.7 (61.7–67.5)	0.01 (1)	0.92
Journals (academic)	160	58.0 (51.9–63.9)	116	42.0 (36.1–48.1)	276	25.7 (23.1–28.4)	4.7 (1)	0.03
Newspaper	52	43.3 (34.3–52.7)	68	56.7 (47.3–65.7)	120	11.2 (9.3–13.2)	4.4 (1)	0.04
Discussion with coworkers	303	50.7 (46.6–54.7)	295	49.3 (45.3–53.4)	598	55.6 (52.5–58.6)	1.5 (1)	0.22
CDC	365	53.1 (49.3–56.9)	322	46.9 (43.1–50.7)	687	63.8 (60.9–66.7)	0.50 (1)	0.48
Health department	170	52.5 (46.9–58.0)	154	47.5 (42.0–53.1)	324	30.1 (27.4–33.0)	0.004 (1)	0.95
Social media	182	48.4 (43.2–53.6)	194	51.6 (46.4–56.8)	376	34.9 (32.1–37.9)	3.6 (1)	0.06
Total	563	52.3 (49.3–55.3)	513	47.7 (44.7–50.7)	1076	100	-	-

The majority of the samples (55.5%) did not have any of the listed comorbidities (hypertension, diabetes, COPD, asthma, or CHF). Out of the remaining participants, the majority had one comorbidity (31.9%), followed by two comorbidities (10.6%), and three or more comorbidities (2.1%). The number of comorbidities was not significantly associated with vaccine acceptance (P=0.07). Other variables significantly associated with getting the vaccine in descriptive analyses are presented in Table 2. 

Most common reasons for refusing the vaccine were unknown side effects (88.5%), followed by the newness of the vaccine (86.5%), and being unsure of effectiveness (50.1%; Table 3). Other reasons that were not listed on the survey but were mentioned in the comments included concerns about having an allergic reaction (1.2%) or effects on fertility or pregnancy (2.5%).

**Table 3:** Reasons for refusing COVID-19 in a large sample of healthcare workers in rural Appalachia among 13 facilities (N=513)* *Participants can select multiple answers, proportions do not add up to 100%.

	n	P (95% CI)
The vaccine is relatively new	444	86.5 (83.3–89.4)
Unsure if effective	257	50.1 (45.7–54.5)
Unknown side effects	456	88.9 (85.8–91.5)
Unknown origin of vaccine	177	34.5 (30.4–38.8)
Waiting for someone else to take the vaccine	172	33.5 (29.5–37.8)
Been COVID-19 positive	46	9.0 (6.6–11.8)
Afraid of getting COVID-19 from vaccine	67	13.1 (10.3–16.3)
COVID-19 is not as serious as portrayed	14	2.7 (1.5–4.5)
Religious reasons	34	6.6 (4.6–9.1)
Do not believe in vaccines	17	3.3 (1.9–5.3)
Healthcare reasons (immunosuppressed)	38	7.4 (5.3–10.0)
High-risk individuals at home	58	11.3 (8.7–14.4)
Cost	8	1.6 (0.7–3.0)
Insurance reasons	7	1.4 (0.6–2.8)
Total	513	100

The final multivariable model included age, gender, occupation, diabetes, previous COVID-19 positive test, and getting flu vaccine yearly (Table 4). The odds of accepting the vaccine was higher for older workers (OR and 95% CI: 1.05, 1.04-1.06). Females had 50% lower odds of accepting the vaccine compared with males (OR and 95% CI: 0.50, 0.35-0.73). Physicians had the highest odds of accepting the vaccines, with significantly higher odds of receiving the vaccine compared to registered nurses (RNs), nurse practitioners (NPs), and nurse externs (OR and 95% CI: 3.24, 1.53-6.86) and compared to allied health professionals (OR and 95% CI: 4.0, 1.38-11.53). Those who were categorized in the other category (administration, information technologist [IT], etc.) had the lowest odds of being vaccinated, but were only significantly different from physicians (OR and 95% CI: 0.25, 0.08-0.71). Those with previous positive COVID-19 test and those with diabetes were less likely to receive the vaccine (ORs and 95% CIs: 0.59, 0.39-0.89 and 0.50, 0.31-0.80, respectively). Finally, those who receive the yearly flu vaccine had over six times the odds of receiving the COVID-19 vaccine compared to those who do not take the yearly flu vaccine (OR and 95% CI: 6.16, 3.82-9.95).

**Table 4:** Mixed-effects logistic regression model results for predicting COVID-19 acceptance in a large sample of healthcare workers in rural Appalachia among 13 facilities (N=995)* Random intercept variance estimate (95% CI) for location: 0.09 (0.03–0.34). *Excluded observations with missing data. †Includes physicians, anesthesiologists, and CRNAs. ‡Includes administration, housekeeping, IS, IT, maintenance, and other miscellaneous positions. RN: registered nurse, NP: nurse practitioner.

Variable	OR	95% CI	p-value
Age (years)	1.05	1.04–1.06	<0.001
Gender			
Male (ref)	-	-	
Female	0.50	0.35–0.73	<0.001
Occupation			
RN, NP, and nurse externs (ref)	-	-	
Physicians†	3.24	1.53–6.85	0.002
Allied health	0.81	0.56–1.18	0.28
Pharmacists and pharmacy technicians	1.12	0.58–2.15	0.75
Other^‡^	0.79	0.55–1.14	0.22
Diabetes	0.59	0.39–0.89	0.01
Had positive COVID-19 test	0.50	0.31–0.80	<0.01
Takes flu vaccine yearly	6.16	3.82–9.95	<0.001
Intercept	0.07	0.03–0.16	<0.001

## Discussion

Vaccination is crucial during the COVID-19 pandemic to reduce infection rates and deaths. Vaccine’s effectiveness is dependent on the formation of a long-standing immune response and the proportion of people willing to accept the vaccine. Through our survey-based study, we have identified characteristics of healthcare workers who are more likely to refuse the vaccine and have identified the likely reasons for vaccine refusal in the rural Appalachia healthcare population. With the demographics of a rural setting, poverty, increased comorbidities, and low education attainment levels, those who live in rural Appalachia rely heavily on healthcare workers’ advice. By focusing on why healthcare workers are hesitant to accept the vaccine, further education of healthcare workers may help increase the percentage of rural Appalachians who get the vaccine.

In our study, we found that 52.3% were willing to accept the vaccine. In comparison, a study done by Reiter et al. of 2006 participants aged 18 or older found that 69% of participants were willing to get the vaccine (48% definitely willing and 21% probably). The study by Reiter et al. was done based on the US general public in March 2020 while our study was based on the rural Appalachia area in Eastern Kentucky and a part of Western Virginia in December 2020 when news of the COVID vaccines were in the headlines [11]. This difference may be attributed to the difference in demographics between the general US population surveyed and the healthcare population specific to rural Appalachia. Also, our survey was conducted just prior to initial vaccine administration and more information about the specific COVID-19 vaccines was available, which may impact acceptance. When compared to similar studies conducted among healthcare workers in the Dominican Republic of the Congo, our study resulted in a higher acceptance rate compared to the 28% acceptance rate in the Dominican Republic of the Congo [13]. Reversely, our study had a lower acceptance rate when compared to a recent similar study conducted in France which noted an acceptance rate of 75% among healthcare workers surveyed [14]. This difference may be attributed to study demographics particularly in relation to occupation. The study done in France, for example, saw a larger proportion of the respondents as physicians and pharmacists which likely contributes to their high acceptance rates [14].

Higher age was associated with greater acceptance of the vaccine. In the studies mentioned previously done in the Dominican Republic of Congo and France, similar results were noted [13,14]. This may be due to fact that the prognosis of COVID-19 infection is worse in older adults due to increased comorbidities, and hence, they are more willing to receive the vaccine to prevent COVID-19. Our study also found that females had a lower chance of accepting the vaccine in comparison to males even though 31% of our healthcare workers who are women work with COVID patients daily out of whom 14% were diagnosed as COVID positive. This is likely attributed to the fact that the occupation most resistant to the vaccine were RNs, NPs, and nurse externs who are predominantly female within our healthcare system. A similar trend was observed in the recent study done in France as well. The only comorbidity significantly associated and included in the multivariate model was diabetes which was associated with refusal of the vaccine. None of the other comorbidities were significantly associated with vaccine acceptance in the multivariable model which is interesting given that increased comorbidities are associated with worse prognosis. Surprisingly, increased exposure to COVID-19 was not significantly associated with vaccine acceptance/refusal. As expected, those who previously got COVID-19 were less likely to accept the vaccine because they believe that they already have immunity against COVID-19 (although it is unclear how long innate immunity would last). Also, those who receive the flu vaccine regularly have six times greater odds of receiving the COVID-19 vaccine which may reflect their attitude towards vaccines in general.

When considering reasons for refusing the vaccine, the two most common reasons were “Newness of the vaccine” and “Unsure side-effects.” It is expected that the vaccine is relatively new would be a barrier to vaccine acceptance. Historically, newer vaccines are often less likely to be accepted. The Flu vaccine, which has been around for many decades, has an acceptance rate of 49% in healthcare personnel in 2008, which has increased to 80.6% in the 2019-20 season [6]. This indicates that vaccine acceptance may increase with time. “Unsure side-effects” was the most common reason reported for vaccine refusal. Future studies assessing side effects among those who have received the vaccine are likely to help address this issue. In a study done by Anderson et al., there were no reported serious side-effects within seven days of vaccination although there were non-specific side-effects with more frequent pain at the injection site, fatigue, chills, myalgia, headache, and rarely arthralgia, which mainly occurred after the second dose of the vaccine [15]. The CDC’s V-safe initiative, an app that monitors side-effects among those who have received the vaccine is an important data source for future research in this area [16]. Uncertainty on vaccine efficacy is another reason cited by respondents. The two FDA-approved vaccines have an efficacy of 95% and 94.5% based on previous studies. In order to adequately convey the strong efficacy of the vaccines, healthcare personnel may need more training on vaccine education, which can improve vaccine acceptance. In this study, 172 respondents (33.5%) who refused the vaccine chose “waiting for someone else to take the vaccine first to see what happens.” Further information/education regarding the vaccine directed towards this group, especially as we progress through the phases, can improve vaccine acceptancy.

Among the sources of information as listed in our survey, it appears that those who received their information from journals/newspaper sources were more likely to accept the vaccine. This poses an important point of discussion when it comes to vaccine acceptance. Medical professionals primarily read journals, so in order to affect vaccine acceptance in the public, it is even more crucial that healthcare workers be provided with appropriate education material to present to their patients. A new technique known as motivational interviewing has improved vaccine acceptance by educating patients on the vaccine itself to improve the likelihood of getting the vaccine [17]. Another conversation starter is AIMS, which stands for announcing the vaccine, inquiring about patient’s views, mirrors, and security [17]. Both techniques involve the provider understanding the reasons for hesitancy in order to focus their discussion to further educate the patient to secure a trust from the patient that the information being discussed is important for their better health.

It is also important that key findings of important research be more readily available to the public through more utilized information platforms. With the advances in technology, social media has played a pivoting role during the pandemic. Information is easily published to reach a broad audience. With social marketing, people can be informed of the value of being vaccinated against COVID-19 including the protection that it offers [17]. Social marketing can also help promote others to follow suit.

Limitations

This study did have limitations with more women involved in the study compared to men. Among more than 6000 individuals working among the surveyed hospitals, only 1076 had fully completed the survey. This low response rate may affect the generalizability of the survey as those who responded to the voluntary survey may be different than the underlying population of healthcare workers. In addition to electronic surveys, paper surveys were utilized only among sites where investigators worked. This may result in some selection bias. Additionally, the survey did not present a comprehensive list of comorbidities to choose from. Certain key comorbidities were missing including coronary artery disease, obesity, and chronic kidney disease. The responses provided by participants in the ‘other comorbidities’ section, however, were too varied to be utilized. This may have resulted in underreporting of comorbidities in the study population.

Of note, this study was done before vaccine administration began. Future studies could address ways to improve vaccine acceptance of not only healthcare workers but also the public. Our healthcare employees started getting vaccinations in December 2020 and then completed the vaccination series in January 2021. All employees who were employed by our healthcare system regardless if they were working from home were eligible to come to the hospital to get their vaccination. By February 2021, the percentage of employees vaccinated was 48%, which was lower than what we expected. By March 2021, the percentage of vaccine acceptance increased to 55%. We have no other data source that compares the vaccine acceptance rate in healthcare workers from the surrounding area outside of our hospital system. A factor that could have played a role was waiting for others to take the vaccine first. This gave employees at least two months to gather information about the side effects of the vaccines from the first wave of healthcare employees who were vaccinated leading to a steady increase. In order to increase the numbers of healthcare workers being vaccinated, the hospital system sent at least weekly emails reminding employees when they can be vaccinated and how to sign up. At times, there was a system-wide announcement that it was vaccine day and encouraged those who have not been vaccinated to do so. Then in April 2021, the hospital system started an incentive program that correlated a set total percentage of employees vaccinated within a hospital will get a monetary bonus. The set goal was a 70% vaccination rate within a hospital site. As the vaccination rate exceeds the set goal, the bonus value did increase as well. This incentive program likely increased the vaccination rate to 59% since the number of those who decided to then take the vaccine increased steadily after the incentive program was advertised.

## Conclusions

Around 52% of healthcare workers surveyed had responded yes to our survey to get the COVID-19 vaccine. The effectiveness of the COVID-19 vaccine in preventing further COVID-19-related deaths is heavily linked to vaccine acceptance among the public. Now that we are progressing through the vaccination phases, it is crucial to address the concerns of healthcare workers regarding the vaccine through further research and targeted educational resources, which will likely lead to a greater acceptance rate among healthcare workers and subsequently increase public acceptance through recommendation. Based on the vaccination rate, our healthcare workers did eventually surpass our estimation likely secondary to those who were waiting for others to get the vaccine first. Our vaccination rates improved after offering an incentive. Intuitive strategies are needed to increase vaccination rates and engage the public in discussions regarding the vaccine.

## Appendices

COVID 19 vaccine questionnaire

This survey is confidential. You do not have to share your name or any other identifier.

Disclaimer: Participation in this survey is on a voluntary basis. The Survey Monkey does not contain any identifiers and it cannot be traced to any individual. The results of the survey are for educational purposes only. The investigators do not derive any monetary benefit from this questionnaire. There are no benefits or compensation to the participants for completing the questionnaire.
 

Are you willing to answer a few questions?

Yes or No

1. Are you willing to take the COVID 19 vaccine?

Yes or No

If No, why? (Select any of the below)

The vaccine is relatively new

Unsure if the vaccine is effective

Unknown side effects of the vaccine at this time

Unknown origin of where the vaccine was made

Waiting for someone else to take the vaccine first to see what happens

Been (+) for COVID 19 already

Afraid of getting COVID 19 from the vaccine

COVID 19 is not as serious as portrayed

Religious reasons

Do not believe in vaccines

Healthcare reasons (immunosuppressed)

High-risk individuals at home

Cost

Insurance reason

Other (please specify)

2. Have you tested positive for COVID 19? 

Yes or No

3. What has been your exposure to COVID 19 at the workplace?

Not working with COVID patients

Working with COVID patients daily

Working with COVID patients weekly

Working with COVID patients monthly

4. What is your age?  ____

5. What is your gender? 

Male or Female

6. What is your ethnicity? 

Caucasian

African American

Asian

Hispanic or Latino

Native American

Other (please specify):  

7. What is your current occupation?

Physician

Please specify department (IM, FM, peds, surgery, etc):

Physician assistant

Please specify department (IM, FM, peds, surgery, etc):

Nurse practitioners 

Please specify department (IM, FM, peds, surgery, etc):

Registered nurses

Physical/occupational/speech therapists

Respiratory therapists

Radiologic technicians (X-ray, CT, ECHO)

Clinical laboratory technicians and phlebotomists

Pharmacists/pharmacist technicians 

Surgical technicians

Administration

Medical Assistants

Anesthesiologists/CRNA

Nursing aides, psychiatry aides, home health aides

Dietitians and nutritionists

Allied health professions (phlebotomists, medical laboratory scientists, dieticians, social workers)

Medical records, admission, QI

Housekeeping

Other:

8. Which hospital are you primarily working at? 

9. Where do you work primarily? (Select all that apply)

Hospital 

Ambulatory (clinic)

Emergency department

10. Do you have any of the following comorbidities? 

Hypertension

Diabetes

COPD

Asthma

CHF

None

Other: (please specify)

11. Do you normally get the flu vaccine every year?

Yes or No
 

12. If given the option, will you recommend your family members to get the COVID 19 vaccine? 

Yes or No

13. Where have you been getting information about the COVID 19 vaccine? 

Television - news

Internet

Journals (academic)

Newspaper

Discussion with co-workers

CDC

Health department 

Social media 
